# The Role of Methylation as an Epigenetic Marker in HPV‐Related Oral Lesions

**DOI:** 10.1002/jmv.70459

**Published:** 2025-06-24

**Authors:** Michela Buttà, Nicola Serra, Arianna Sucato, Daniela Cabibi, Giuseppina Campisi, Vera Panzarella, Giulia Alfedi, Daniela Pistoia, Giuseppina Capra

**Affiliations:** ^1^ Department of Health Promotion, Mother and Child Care, Internal Medicine, Medical Specialties G. D'Alessandro University of Palermo Palermo Italy; ^2^ Department of Neuroscience, Reproductive Sciences and Dentistry‐Audiology Section University of Naples Federico II Naples Italy; ^3^ Anatomic Pathology Unit University Hospital “P. Giaccone” Palermo Italy; ^4^ Unit of Oral Medicine and Dentistry for Fragile Patients, Department of Rehabilitation Fragility, and Continuity of Care University Hospital “P. Giaccone” Palermo Italy; ^5^ Department of Biomedicine, Neuroscience and Advanced Diagnostics (BiND) University of Palermo Palermo Italy; ^6^ Department of Precision Medicine in Medical, Surgical and Critical Care (MePreCC) University of Palermo Palermo Italy; ^7^ Fujirebio Italia S.r.l. Roma Italy; ^8^ Microbiology and Virology Unit University Hospital “P. Giaccone” Palermo Italy

**Keywords:** DNA methylation, human papillomavirus (HPV), molecular biomarkers, oral cancer, oral infection

## Abstract

Oral and oropharyngeal cancers, caused by persistent human papillomavirus (HPV), have recently increased. Diagnostic methods often fail to assess precancerous lesion risk, delaying oral cancer diagnosis. New molecular biomarkers, particularly DNA methylation, are sought to better stratify patients' risk. The PreCursor‐M+ (Fujirebio, Tokyo, Japan), which analyze hypermethylation of the two onco‐suppressor *FAM19A4* and *miR124‐2* in cervical samples from high‐risk HPV‐positive women, was used to assess the methylation level of 111 oral samples distinguished in oral squamous cell carcinomas (OSCC), oral potentially malignant disorders (OPMD) benign lesions (BL), and no lesions (NL). HPV was detected by INNO‐LiPA HPV Genotyping Extra II (Fujirebio, Tokyo, Japan). Hypermethylation was correlated with the severity of the diagnosis. A positive result was more common in OSCC (*p* < 0.0001). HPV positivity correlated with hypermethylation in OSCCs (32.4%, *p* = 0.0006), although statistical significance was also found in HPV‐negatives (*p* = 0.0007). HPV16‐positive OSCC showed higher methylation. Targets' methylation increased from the NL to the BL, OPMD and OSCCs groups. The methylation status of *FAM19A4* and *miR‐124‐2* may play an important role in the progression of oral cancer and, consequently, in determining the prognosis of patients with OPMD, for whom hypermethylation would suggest the need for close monitoring. Furthermore, HPV16's association with hypermethylation suggests its involvement in oral carcinogenesis. To confirm these results and gain further insight into HPV's role in methylation impairment, the sample size will be increased.

## 1 Introduction

Head and neck squamous cell carcinomas (HNSCCs) are the seventh most common malignancy worldwide [1]. These heterogeneous neoplasms arise in various upper aerodigestive tract sites, with oral and laryngeal cancers being the most prevalent [2, 3]. While HNSCCs have traditionally been associated with tobacco and alcohol, persistent HPV infection is now recognized as a major factor in the rising rates of oral squamous cell carcinoma (OSCC) and oropharyngeal squamous cell carcinoma (OPSCC) [4, 5, 6, 7]. Human papillomavirus (HPV), the most common sexually transmitted pathogen [8], infects basal keratinocytes. A persistent infection for more than 12–24 months puts the patient at risk of developing benign and malignant hyperproliferative lesions [9]. In particular, the International Agency for Research on Cancer (IARC) has distinguished the genotypes, based on their association with the development of malignant lesions, into high‐risk (hrHPV) and low‐risk (lrHPV). Cervical cancer is strongly linked to HPV (99.7%) [10]; while other anogenital cancers show lower but significant associations: anal (88%) [11], vulvar (43%) [12], vaginal (70%) [13], and penile (50%) [14]. HPV's role in HNSCC is less clear, with prevalence varying from 20% to 80%, and mainly involving HPV16 [15, 16]. In particular, HPV is a known risk factor for oropharyngeal cancer (20%–70% of cases) [17], with less certain links to oral (0%–37%) and laryngeal (23%–33%) cancers [18, 19, 20, 21, 22]. Differentiating HPV‐positive and HPV‐negative HNSCCS, especially oropharyngeal cancers, is crucial since HPV‐positive oropharyngeal cancers have a much better prognosis (79% survival rates) than HPV‐negative ones (31%) [5, 7].

Oral cancer development can be preceded by oral potentially malignant disorders (OPMDs), including leukoplakia and erythroplakia, with malignant transformation influenced by dysplasia [23, 24, 25]. HPV's role in OPMD is unclear.

Benign oral lesions, such as condylomas and papillomas, are traditionally linked to lrHPV genotypes, primarily HPV 6 and 11 [22, 26]. When papillomas grow along the respiratory tract, this is called recurrent respiratory papillomatosis (RRP) or laryngeal papillomatosis, as the larynx is the most common site of involvement [27].

Clinical and pathological examinations are not always effective in delineating the precise risk of malignant progression of pre‐cancerous lesions, resulting in frequent late diagnosis of oral cancer, and leading to a poor prognosis for many patients.

In this scenario, identifying new molecular biomarkers with diagnostic and/or prognostic value could aid in improving early diagnosis, thus leading to an adequate diagnosis, improved treatment, and better patient survival and quality of life [25]. In recent decades, the search for new ways to identify and characterize the progression towards the cancer phenotype has focused on epigenetic mechanisms, of which DNA methylation is one of the most widely characterized and a well‐known HPV‐hampered mechanism.

The quest for methylation biomarkers in HPV‐positive cancers has mainly focused on preneoplastic and neoplastic lesions of the cervix. Indeed, numerous studies have led to the identification of several specific genes (i.e., *miR‐124, EPB41L3, CADM1, PAX1*, and *FAM19A4*) whose abnormal hypermethylation is associated with high sensitivity to cell premalignant and malignant phenotype and which could then serve as prognostic and diagnostic biomarkers [28, 29, 30, 31, 32]. The hypermethylation of these targets is linked to cervical cancer progression and poor prognosis [29, 33, 34, 35, 36, 37]. Recent studies suggest *FAM19A4* hypermethylation and *miR124‐2* silencing also in oral cancers [38, 39]. Whole genome bisulphite sequencing (WGBS) analyzes performed on HPV‐negative and HPV‐positive oral cancer specimens have shown statistically significant hypermethylation of *FAM19A4* promoter in neoplastic lesions compared to normal adjacent tissue. *MiR124‐2*, besides being methylation‐dependently silenced in a plethora of cancers [40, 41], can downregulate in vitro the expression of integrin beta‐1 (ITGB1), involved in adherence processes and motility of OSCC cells, hence reducing invasive and migrating genotype [42].

This study is the first to investigate the association between *FAM19A4* and *miR124‐2* hypermethylation in HPV‐positive patients with potentially malignant or neoplastic lesions. We aim to evaluate the potential of these epigenetic markers as prognostic indicators.

## 2 Materials and Methods

### 2.1 Patient Recruitment and Sample Collection

One hundred and twenty‐three patients with suspected HPV‐related oral lesions were recruited from the Oral Medicine and Dentistry for Fragile Patients Unit at the University Hospital “P. Giaccone” of Palermo. To collect samples for HPV analysis, each patient self‐administered a 10 mL oral rinse using Original Mint Scope mouthwash (Procter & Gamble), following the procedure described in Buttà et al. [43]. Rinse samples were sent to the hospital's Microbiology and Virology Unit, while biopsies were sent to the Pathological Anatomy Unit for histological examination. This examination classified lesions as oral squamous cell carcinoma (OSCC), OPMD, or benign lesions (BL). Sample selection was based on two different criteria: the availability of a histological diagnosis of SCC, potentially malignant lesions (erythroplakia, leukoplakia, and lichen planus) and BL (papillomas), and the need to have an equal number of HPV‐positive and negative patients in each group. To these, it was added a group of healthy individuals who did not show oral lesions (No lesion= NL), to whom, then, a biopsy was not performed. This group also consisted of approximately the same proportion of HPV‐positive and negative individuals. Patients belonging to every group did not have any other chronic diseases or cancers: we specifically excluded those patients showing other ailments of the oral cavity which could affect hypermethylation level. The ethical committee board of the University Hospital Policlinic, P. Giaccone, Palermo approved the study (approval numbers #03/2013 and #04/2024).

### 2.2 Sample Processing

Cellular components were isolated by centrifugation at 1600 rpm for 10′, resuspended in 1–3 mL of phosphate‐buffered saline (PBS), based on yield, and dispensed into 1.5 mL Eppendorf tubes (1 mL each). The obtained material was centrifuged again at 13,000 rpm for 5 min, and after the removal of the supernatant, each pellet was stored at −20°C or processed immediately.

### 2.3 DNA Extraction and HPV Genotyping

DNA extraction was carried out using the Elitech Ingenius automated system (Elitech Group, Turin, Italy). The DNA concentration was quantified using a Qubit fluorometer (Thermofisher Scientific, Waltham, Massachusetts, USA). HPV detection and genotyping was performed with the CE‐IVD INNO‐LiPA HPV Genotyping Extra II diagnostic kit (Fujirebio, Tokyo, Japan), which allows the identification of 32 distinct genotypes, distinguished in 12 lrHPV (HPV6, HPV11, HPV40, HPV42, HPV43, HPV44, HPV54, HPV61, HPV62, HPV81, HPV83, and HPV89) and 20 types classified as hrHPV (HPV16, HPV18, HPV31, HPV33, HPV35, HPV39, HPV45, HPV51, HPV52, HPV56, HPV58, HPV59, HPV67, HPV68, HPV26, HPV53, HPV66, HPV70, HPV73, and HPV82). The reaction was carried out on the thermocycler GeneAmp PCR System 9700 (Applied Biosystems, Thermofisher Scientific, Waltham, Massachusetts, USA) using the following PCR cycling profile: decontamination from uracil containing DNA at 37°C for 10 min, denaturation at 94°C for 9 min, denaturation at 94°C for 30 s, primers annealing at 52°C for 45 s, primers extension at 72°C for 45 s (steps from the third to the fifth were repeated 40 times), hold at 72°C for less than 2 h.

Since SPF10‐based primers can amplify more than 54 HPV genotypes, some samples testing positive only for the HPV control probes were amplified with PGMY09/PGMY11 and GP05+/GP06+ primers as described elsewhere [44] and subsequently subjected to Sanger sequencing on an ABI Prism 3130 Genetic Analyzer (Thermo Fisher Scientific, Waltham, MA). Genotypes were then obtained by BLAST sequence alignment.

### 2.4 Methylation Analysis

Methylation analysis required initial treatment with sodium bisulphite, performed with the EZ DNA Methylation kit (Zymo Research, Irvine, USA) according to the manufacturer's instructions.

During this procedure, the bisulphite acts on the unmethylated cytosines by deaminating them into uracils, while leaving the methylated cytosines intact. Following amplification by PCR, the former will correspond to thymines.

In order for the conversion to take place correctly, 200 ng of DNA was required, so the corresponding volume was calculated accordingly.

For all samples that produced enough DNA, 200 ng of DNA was used for conversion. In contrast, for samples that had concentrations below the minimum acceptable, an attempt was made to add the maximum amount of ng in conversion by taking 45 µL of extracted DNA.

Following the conversion process, the converted DNA samples were subjected to the real‐time multiplex qPCR of the PreCursor‐M+ kit (Fujirebio, Tokyo, Japan). The method involves the use of primers designed to couple only to the originally methylated DNA of the two target regions corresponding to the promoter sequences of the *FAM19A4* and *miR124‐2* genes. In addition, a third pair of primers allows the amplification of β‐actin (β‐Act), which checks the quality of the converted and methylated DNA.“TaqMan” fluorescent hydrolysis probes and a standard concentration calibrator of the three targets are also used.

The qPCR was performed on the magnetic induction cycler MIC IVD qPCR Cycler (Bio Molecular Testing, Upper Coomera, Queensland, Australia) using the following PCR cycling profile: denaturation at 95°C for 5 min, denaturation at 95°C for 15 s, annealing and extension at 63°C for 60 s (second and third steps were repeated 40 times).

### 2.5 Real‐Time PCR Results Analysis

The quantitative evaluation required the initial normalization of the two target genes compared to the endogenous control (β‐Act) by calculating the Δ*Ct*, that is, the difference between the target's *Ct* and the control's *Ct*. The value of Δ*Ct* was then used to evaluate the methylation level using the ΔΔ*Ct* method:

$$\Delta \Delta Ct=\Delta Ct_{\mathrm{sample}}-\Delta Ct_{\mathrm{calibrator}}.$$

The comparison between the ΔΔ*Ct* values with the cervical cut‐offs provided by the diagnostic kit allowed the identification of two results, that is, “absence of hypermethylation” or “presence of hypermethylation.” More specifically, the absence of hypermethylation corresponded to ΔΔ*Ct* values of both targets higher than the cut‐off defined for cervical samples (i.e., *FAM19A4* > 9.66 and *miR124‐2* > 6.0); while the presence of hypermethylation was defined by ΔΔ*Ct* value of at least one of the two targets lower than the cut‐offs defined for cervical samples (i.e., *FAM19A4* ≤ 9.66 and/or *miR124‐*2 ≤ 6.0). The use of cervical cut‐offs was imposed by the fact that cut‐offs specific to oral samples are not currently available.

To overcome this limit, at the same time, we performed another evaluation, namely the Livak's method, using the 2^‐ΔΔ*Ct*^ calculation formula. This approach allowed quantifying methylation as “% of methylated cells in the sample,” without, thus, the need of a cut‐off. This percentage is distributed on a scale ranging from 0 (unmethylated) to values above 0 (different levels of hypermethylation, depending on the value). This formula indicates how often the gene is methylated in the test compared to the calibrator, which normalized the data to the housekeeping gene.

Graphics reporting the methylation results were all generated by Microsoft Excel.

### 2.6 Statistical Analysis

For categorical variables, data are presented as numbers and percentages, and continuous data are expressed as mean ± standard deviation (SD) unless otherwise specified.

A binomial test was performed to compare two mutually exclusive proportions or percentages in groups.

The multicomparison chi‐square test was used to define significant differences among groups. If the chi‐square test was positive (*p* < 0.05), residual analysis with continuity correction for *Z*‐test was performed to localize the highest or lowest significant presence. In addition to the analyzes between three or more modalities of a variable, the chi‐square goodness of fit was used.

Test for normal distribution was performed by the Shapiro–Wilk test.

One‐way analysis of variance (ANOVA) test was used to test the difference between the means of several subgroups of a variable. If the ANOVA test was positive (*p* < 0.05) then the post hoc test was used by Scheffé's method for pairwise comparison of subgroups. Before the ANOVA test, Levene's Test for Equality of Variances was performed. If the data was not normally distributed, the Kruskal–Wallis test was performed in multi‐comparison among three or more unpaired samples. If the Kruskal–Wallis test was significant (*p* < 0.05), the Conover post hoc test was used for pairwise comparison of subgroups.

The relationship between Infections and other parameters was calculated using the chi‐square test or Fisher's exact test (dichotomous vs. dichotomous), or the Mann–Whitney test (dichotomous vs. no normal continuous data). For this step, we define the following variables:

- Diagnoses: NL = 0, BL = 1, potentially malignant lesions = 2, cancer lesions = 3 (the scale was defined according to the severity of the disease).
- Age.
- Sex: male = 1, female = 0.
- Genotype risk: hr or hr/lr = 1, lr = 0; HPV infected: yes = 1, no = 0.
- Hypermethylation: absence = 0, presence = 1.

Multiple linear regression was used to find the best‐fitting model to describe the relationship between the dichotomous characteristic of interest (the dependent variable: diagnoses) and a set of independent variables such as hypermethylation, age, sex, genotype risk, HPV infection, and the number of genotypes. Additionally, since some statistical tests include small data, we performed the power analysis for each statistical test using the effect size. Notably, the effect size was computed by phi coefficient for categorical variables, by *η* ^2^ and *r* for nonparametric test (Mann–Whitney test, Kruskal Wallis test, and Wilcoxon signed‐rank test, respectively), while for parametric test, the *η* ^2^(One way ANOVA test) and Cohen's *d* index were used (paired and unpaired *t*‐test).

All tests with *p* < 0.05 were considered significant. All data were analyzed using the Matlab statistical toolbox version 2008 (MathWorks, Natick, MA, USA) for 32‐bit Windows.

## 3 Results

The cohort consisted of 123 patients, 12 of whom were excluded due to insufficient DNA, leaving 111 samples. Of this final group, 60.4% (67/111) were men and 39.6% (44/111) were women, with an overall mean age of 55.7 (±16.2).

The percentage of HPV‐positive samples was 48.6% (54/111), of which 81.5% (44/54) were single infections, and 64.8% (35/54) displayed at least one oncogenic HPV type (Supporting Information S1: File S1).

73.9% (82/111) of participants agreed to give us information about their smoking habits and alcohol consumption. Of those who responded, 42.7% (35/82) were smokers, and 26.8% (22/82) drank alcohol. No statistically significant association was found between smoking and hypermethylation (*p* = 0.46), or between alcohol consumption and hypermethylation (*p* = 0.79). The cohort was subdivided into four groups depending on the diagnosis.

Each group was composed as follows (Supporting Information S1: File S1):

- NL group: 20 patients with NL, with 45% (9) males and 55% (11) females, with a mean age of 39.2 y.o.;
- BL group: 37 patients with BL, with 59.5% (22) males and 40.5% (15) females, with a mean age of 51.1 y.o.;
- OPMD group: 17 patients with potentially malignant lesions, with 52.9% (9) males and 47.1% (8) females, with a mean age of 66.4 y.o.;
- OSCC group: 37 patients with carcinoma, with 73% (27) males and 27% (10) females, with a mean age of 64.5 y.o.

Supporting Information S1: File S1 also shows the presence or absence of hypermethylation according to the cut‐offs for cervical lesions.

A total of 29 genotypes were identified; the prevalent types were HPV16, with a rate of 25.9% (14/54), HPV6, with 14.8% (8/54), and HPV66, with 12.9% (7/54).

In Table 1, we reported the clinical information among patients with NL, BL, OPMD, and OSCC. NL patients were significantly younger than BL (39.2 vs. 51.1, *p* < 0.05), OPMD (39.2 vs. 66.4, *p* < 0.05), and OSCC patients (39.2 vs. 64.5, *p* < 0.05).

**Table 1:** Characteristics and comparison among patients belonging to the four groups.

Parameters	NL	BL	OPMD	OSCC	Analysis among groups	
	*n* = 20	*n* = 37	*n* = 17	*n* = 37	*p*‐value (test)	Effect size
Age					*p* < 0.001* (A) NL < BL, *p* < 0.05* (Sh) NL < OPMD, *p* < 0.05* Sh) NL < OSCC, *p* < 0.05* (Sh) BL < OPMD, *p* < 0.05* Sh) BL < OSCC, *p* < 0.05* (Sh)	*η* ^2^ = 3.80 large effect
Mean ± SD	39.2 ± 9.8	51.1 ± 15.6	66.4 ± 10.6	64.5 ± 12.3		
Median (IQR)	41 (31.5, 48)	53 (40, 60.8)	68 (57.5, 74)	66 (54.8, 73.3)		
Sex					*p* = 0.18 (C)	Phi = 0.209 Low effect
Male	45% (9)	59.5% (22)	52.9% (9)	73% (27)		
Female	55% (11)	40.5% (15)	47.1% (8)	27% (10)		
HPV infected	50% (10)	48.6% (18)	41.2% (7)	51.2% (19)	0.92 (C)	Phi = 0.07 Trivial effect
Number of genotypes					*p* = 0.45 (F)	Phi = 0.155 Low effect
One	30% (6)	40.5% (15)	100% (7)	43.2% (16)		
Two	5% (1)	5.4% (2)	0.0% (0)	2.7% (1)		
Three	5% (1)	2.7% (1)	0.0% (0)	0.0% (0)		
More than three	10% (2)	0.0% (0)	0.0% (0)	5.4% (2)		
Genotype risk					*p* = 0.0033 * (F) OSCC‐LR/HR**, *p* = 0.0279 (Z) BL‐LR**, *p* = 0.0048 (Z)	Phi = 0.485 medium effect
lr/hr or hr	90% (9)	38.9% (7)	42.9% (3)	43.2% (16)		
hr	10% (1)	61.1% (11)	57.1% (4)	8.1% (3)		
Hypermethylation in total patients					*p* = 0.0001* (C) NL‐Neg**, *p* = 0.0023 (Z) BL‐Neg**, *p* = 0.0162 (Z) OSCC‐pos**, *p* < 0.0001 (Z)	Phi = 0.492 medium effect
Absent (*n* = 73)	95% (19)	75.7% (30)	58.8% (11)	35.1% (13)		
Present (*n* = 38)	5% (1)	18.9% (7)	35.3% (6)	64.9% (24)		
Hypermethylation in HPV‐negative patients					*p* = 0.0015* (F) NL‐Neg**, *p* = 0.0105 (Z) OSCC‐pos**, *p* = 0.0007 (Z)	Phi = 0.514 medium effect
Absent (*n* = 37)	50% (10)	40.5% (15)	35.3% (6)	16.2% (6)		
Present (*n* = 20)	0.0% (0)	10.8% (4)	23.5% (4)	32.4% (12)		
Hypermethylation in HPV‐positive patients					*p* = 0.0055* (F) OSCC‐pos**, *p* = 0.0006 (Z)	Phi = 0.479 medium effect
Absent (*n* = 36)	45% (9)	40.5% (15)	29.4% (5)	18.% (7)		
Present (*n* = 18)	5% (1)	8.1% (3)	11.8% (2)	32.4% (12)		

*Note:* Percentages were calculated against the number of diagnosis subgroups. Significantly less frequent; F = Fisher's exact test; A = one way ANOVA test; Sh = Scheffé post hoc test for pairwise comparisons; no lesions (NL), benign lesions (BL), oral potentially malignant disorders (OPMD) and oral squamous cell carcinoma (OSCC).

* Significant test; C = chi‐square test; *Z* = *z*‐test.

** Significantly more frequent.

Genotype risk and diagnosis were significantly correlated (*p* = 0.0033), with the OSCC group characterized by a substantially higher rate of hrHPVs, alone or together with lrHPVs in multiple infections (*p* = 0.0279). Conversely, lrHPVs were significantly more frequent in the BL group (*p* = 0.0048).

**Figure 1:**
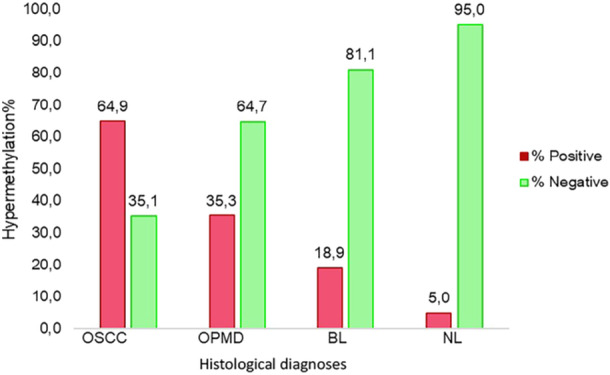
Bar chart describing the percentage of positive (red) and negative (green) samples for hypermethylation based on protocol's cut‐offs in each of the four categories, regardless of HPV positivity. BL, benign lesions; NL, no lesions; OPMD, oral potentially malignant disorders; OSCC, oral squamous cell carcinomas.

Hypermethylation analysis was performed considering the positivity cut‐offs of the kit developed for cervical samples. Presence of hypermethylation, that is, a ΔΔ*Ct* value below the cut‐off for cervical lesions for at least one of the two targets, was significantly more frequent in OSCC (*p* < 0.0001). Absence of hypermethylation, that is, a ΔΔ*Ct* value above the cut‐off designed for cervical lesions for both targets, was significantly more frequent in NL and BL (*p* = 0.0023 and *p* = 0.0162, respectively). The graphical representation of these results is shown in Figure 1, which clearly shows the progressive decrease in the number of samples in which hypermethylation was present from OSCC (64.9%) to OPMD (35.3%), BL (18.9%) and NL (5%), as well as the progressive increase in the number of samples in which hypermethylation was absent from OSCC (35.1%) to OPMD (64.7%), BL (81.1%), and NL (95%).

Looking specifically at the HPV‐positive patients, OSCC was the only group showing a significant positive correlation between HPV‐positivity and hypermethylation (32.4%, *p* = 0.0006). However, HPV‐negative patients in the same group also had a significant presence of hypermethylation (32.4%, *p* = 0.0007).

Similarly, HPV‐negative patients had a significant absence of hypermethylation in the NL group (50%, *p* = 0.0105).

Notably, form power analysis on the significant tests of Table 1, was found large effect size about the relationships between diagnosis variable (NL, BL, OPMD, and OSCC) and age (*η* ^2^ = 3.80), that is, the sample size of the diagnosis groups had a low probability to introduce statistical bias on the results. Instead, for Genotype risk, Hypermethylation in total patients, Hypermethylation in HPV‐negative patients, and Hypermethylation in HPV‐positive patients, we found a medium effect size. In other words, we found a moderate probability of statistical bias on the results due to the sample size of the diagnosis groups. In this case, the results should be confirmed on larger cohorts.

Table 2 shows the association analysis between hypermethylation and age, sex, diagnoses, HPV infection, number of genotypes, and genotype risk. A significant correlation was found between hypermethylation and age (*p* < 0.0001) and hypermethylation and diagnoses (*p* < 0.0001). About the latter, the post hoc *Z*‐test showed that the NL and BL lesions were more frequent among the group showing absence of hypermethylation (26%, *p* = 0.0023; 41.1%, *p* = 0.0162, respectively), while the OSCCs were more frequent in the group showing the presence of hypermethylation (63.2%, *p* < 0.0001) (Table 2). From power analysis, as shown in Table 2, we found a large effect size between hypermethylation and age (*d* = 0.946), indicating a low probability of statistical bias in this result. In contrast, the effect sizes between hypermethylation and gender and hypermethylation and diagnosis were moderate (Phi = 0.22 and 0.493, respectively). In these cases, a moderate probability of statistical bias on the results was found. These results should be confirmed on larger samples.

**Table 2:** Relationships analysis between hypermethylation in all patients and age, sex, HPV infection, symptoms, number of genotypes, and genotype risk.

	Hypermethylation			
Parameters	Negative	Positive	Negative versus positive	
	*N* = 73	*N* = 38	*p*‐value (test)	Effect size
Hypermethylation tot*/Age*	50.9 ± 16.0	65.0 ± 12.2	*p* < 0.0001* (T)	*d* = 0.946 large effect
Hypermethylation tot*/Sex*	42.5% (31 M) 57.5% (42 F)	65.8% (25 M) 34.2% (13 F)	0.40 (C)	Phi = 0.22 medium effect
Hypermethylation tot/Diagnoses	26.0% (19 NL) 41.1% (30 BL) 15.1% (11 OPMD) 17.8% (13 OSCC)	2.6% (1 NL) 18.4% (7 BL) 15.8% (6 OPMD) 63.2% (24 OSCC)	*p* = 0.0001* (C) Negative‐NL**, *p* = 0.0023 (Z) Negative‐BL**, *p* = 0.0162 (Z) Positive‐OSCC**, *p* < 0.0001 (Z)	Phi = 0.492 medium effect
Hypermethylation tot*/HPV infection*	50.7% (37 HPV−) 49.3% (36 HPV+)	52.6% (20 HPV−) 47.4% (18 HPV+)	0.85 (C)	Phi = 0.02 trivial effect
Hypermethylation tot*/*Number of genotypes	Mean rank: 56.0	Mean rank: 55.9	0.98 (MW)	*η* ^2^< 0.0001 trivial effect
Hypermethylation tot*/*Genotype risk	*n* = 36 36.1% (13 lr) 63.9% (23 h or hr/lr)	*n* = 18 33.3% (6 lr) 66.7% (12 h or hr/lr)	0.84 (C)	Phi = 0.03 trivial effect

*Note:* Percentages were calculated against the number of the two groups of samples positive and negative for hypermethylation.

* Significant test (*p* < 0.05), C = chi‐square test, T = unpaired *t*‐test.

** Significantly more frequent; no lesions (NL), benign lesions (BL), oral potentially malignant disorders (OPMD) and oral squamous cell carcinoma (OSCC).

An additional analysis (Supporting Information S2: File S2) was performed to verify the above statistically significant findings regarding the association between age and diagnosis, and hypermethylation and diagnosis in the OSCC group. The aim was to confirm that hypermethylation was not affected by age, focusing specifically on the OSCC group. As shown in Supporting Information S2: File S2, each group considered in this study was stratified into two subgroups: hypermethylation (yes) and hypermethylation (no). Particularly, in the OSCC group, no significant difference by age was observed between the two subgroups, hypermethylation yes and hypermethylation no (65.7 vs. 62.2, *p* = 0.41). This result was also confirmed by power analysis (*d* = 0.29). Therefore, we conclude that the presence of hypermethylation in the OSCC group was not conditioned by age.

The significant variables defined in Table 1 were then analyzed with the disease variable by multiple linear regression analysis, as shown in Table 3. From this analysis, it was found that among age, genotype risk, and hypermethylation, in total patients, age and total hypermethylation were positive predictors of diagnoses (R‐partial = 0.37, *p* = 0.0077; R‐partial = 0.29, *p* = 0.0381, respectively).

**Table 3:** Multiple linear regression analysis between diagnoses and significant variables described in Table 1.

Multiple linear regression	Coefficient	Standard error	*t*	R‐partial	*p*‐value
Multiple correlation coefficient					0.56
Diagnoses/Age	0.03	0.01	2.8	0.37	0.0077*
Diagnoses/Genotype risk	0.30	0.28	1.1	0.15	0.29
Diagnoses/Hypermethylation tot	0.68	0.32	2.1	0.29	0.0381*
Constant	–0.42				

* Significant test.

From multiple linear regression analysis, we found that total hypermethylation was a stronger predictor than variable genotype risk, therefore, we deduce that despite the moderate effect size obtained in Table 1 for total hypermethylation and diagnosis, the result should be considered as a result with low probability of statistical bias.

Specifically considering HPV‐positive samples, an analysis was performed to identify a possible association between the identified genotype, diagnosis, and hypermethylation. In multiple infections, each single genotype was analyzed individually (Table 4).

**Table 4:** Mean and median scores of ΔΔ*Ct* of *FAM19A4* and *mir124‐2* stratified for diagnosis type.

	NL	BL	OPMD	OSCC	Analysis among groups	
					*p*‐value (test)	Effect size
ΔΔCt ratio *FAM19A4*	*n* = 20	*n* = 37	*n* = 17	*n* = 37	*p* < 0.0001* (KW) OSCC > NL*, *p* < 0.05 (Co) OSCC > BL*, *p* < 0.05 (Co) OSCC > OPMD*, *p* < 0.05 (Co) OPMD > NL*, *p *< 0.05 (Co)	*η* ^2^ = 0.19 large effect
Mean ± SD	0.163 ± 0.666	0.102 ± 0.232	0.339 ± 0.573	1.122 ± 1.921		
Median (IQR)	0.0 (0.0, 0.007)	0.0 (0.0, 0.08)	0.03 (0.0, 0.515)	0.28 (0.0, 1.088)		
ΔΔ*Ct* ratio *mir124‐2*	*n* = 20	*n* = 37	*n* = 17	*n* = 37	*p* = 0.0031* (KW) OSCC > NL*, *p* < 0.05 (Co) OSCC > BL*, *p* < 0.05 (Co)	*η* ^2^ = 0.125 medium effect
Mean ± SD	0.236 ± 0.286	0.257 ± 0.583	0.392 ± 0.501	0.92 ± 1.7		
Median (IQR)	0.14 (0.005, 0.34)	0.09 (0.018, 0.253)	0.12 (0.073, 0.72)	0.48 (0.148, 0.155)		

*Significant test; KW, Kruskal–Wallis test; Co, conover post hoc test for pairwise comparisons; no lesions (NL), benign lesions (BL), oral potentially malignant disorders (OPMD), and oral squamous cell carcinoma (OSCC).

In hypermethylated OSCC samples, some genotypes were more frequent than others, namely HPV16, HPV68, and HPV120, but unfortunately, the data were not statistically significant.

To overcome the limitation imposed by the above cut‐offs, the ΔΔ*Ct* ratio (2^−^^ΔΔ*Ct*^) calculation was used to perform a relative quantification of methylation levels, expressed as a percentage.

**Figure 2:**
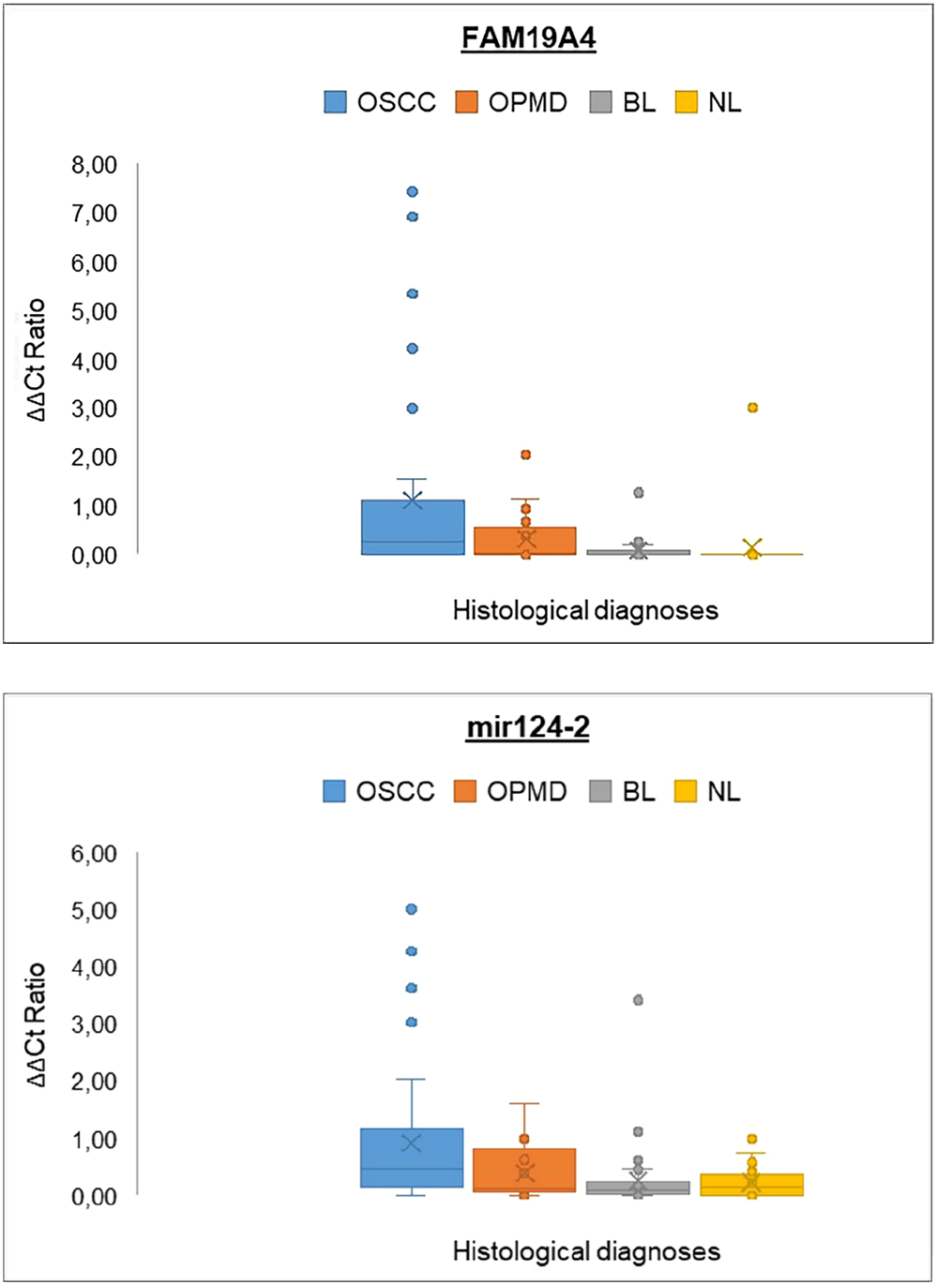
Box plots depicting the ΔΔ*Ct* Ratio values of *FAM19A4* and *miR124‐2* in the four sample categories. BL, benign lesions; NL, no lesions; OPMD, oral potentially malignant disorders; OSCC, oral squamous cell carcinomas.

This evaluation, performed individually for both targets, showed that the methylation levels correlated with the severity of the oral lesion (Figure 2). The statistical analysis performed on the mean and median ΔΔ*Ct* scores of the two targets confirmed this observation (Table 4).

2^−ΔΔ*Ct*^ *FAM19A4* scores were significantly correlated with the diagnosis (*p* < 0.0001), with the post hoc test showing how OSCC score was greater than NL (median: 0.28 vs. 0.0, *p* < 0.05), BL (median: 0.28 vs. 0.0, *p* < 0.05), and OPMD (median: 0.28 vs. 0.03, *p* < 0.05).

A similar result was found for *mir124‐2* 2^−ΔΔCt^ values (*p* = 0.0031), for which the OSCC group score was greater than NL (median: 0.48 vs. 0.14, *p* < 0.05), and BL (median: 0.48 vs. 0.09, *p* < 0.05).

From power analysis, in Table 4, we found a large effect size for the ΔΔ*Ct* ratio of *FAM19A4* and diagnosis (*η* ^2^ = 0.19) and a medium effect size for ΔΔ*Ct* ratio of *mir124‐2* and diagnosis (*η* ^2^ = 0.125).

The same ΔΔ*Ct* ratio scores were used to evaluate the association between hrHPV or lrHPV detected and the diagnosis, as shown in Table 5. From this analysis emerged how *FAM19A4* ΔΔ*Ct* ratio scores varied significantly among lrHPV positive samples, with the OSCC score significantly higher than the BL one (mean: 0.743 vs. 0.083, *p* < 0.05).

**Table 5:** Mean and median scores of ΔΔ*Ct* ratio of *FAM19A4* and *mir124‐2* stratified for diagnosis considering the HPV risk.

HPV+ patients	NL	BL	OPMD	OSCC	Analysis among groups	
					*p*‐value (test)	Effect size
ΔΔ*Ct* ratio *FAM19A4*						
hr/hr‐lr	*n* = 9	*n* = 7	*n* = 3	*n* = 16	*p* = 0.065 (KW)	*η* ^2^ = 0.186 large effect
mean ± SD	0.358 ± 0.988	0.063 ± 0.137	0.0 ± 0.0	1.382 ± 2.503		
Median (IQR)	0.0 (0.0, 0.11)	0.0 (0.0, 0. 005)	0.0 (0.0, 0.0)	0.235 (0.0, 1.1)		
lr	*n* = 1	*n* = 11	*n* = 4	*n* = 3	*p* = 0.027* (A) OSCC > BL*, *p* < 0.05 (Co)	*η* ^2^ = 0.448 large effect
mean ± SD	0.0	0.083 ± 0.140	0.295 ± 0.330	0.743 ± 0.646		
Median (IQR)	0.0	0.0 (0.0, 0.118)	0.25 (0.02, 0.57)	1.06 (0.265, 1.143)		
ΔΔ*Ct* ratio *mir124‐2*						
hr/hr‐lr	*n* = 9	*n* = 7	*n* = 3	*n* = 16	*p* = 0.079 (KW)	
Mean ± SD	0.356 ± 0.364	0.584 ± 1.254	0.107 ± 0.015	0.229 ± 1.542		*η* ^2^ = 0.199 large effect
Median (IQR)	0.14 (0.09, 0.637)	0.13 (0.038, 0.238)	0.11 (0.099, 0.118)	0.62 (0.215, 1.535)		
lr	*n* = 1	*n* = 11	*n* = 4	*n* = 3	*p* = 0.30 (A)	*η* ^2^ = 0.21 large effect
mean ± SD	0.26	0.20 ± 0.32	0.458 ± 0.433	0.677 ± 0.597		
Median (IQR)	0.26	0.07 (0.015, 0.248)	0.38 (0.105, 0.81)	0.9 (0.225, 1.07)		

*Significant test; KW, Kruskal–Wallis test; Co, Conover post hoc test for pairwise comparisons; A = One‐way ANOVA test; Scheffé's test post hoc test for pairwise comparisons; no lesions (NL), benign lesions (BL), oral potentially malignant disorders (OPMD) and oral squamous cell carcinoma (OSCC).

Finally, in Table 5, all tests involving the ΔΔ*Ct* ratio of *FAM19A4* and *mir124‐2* stratified for diagnosis, considering the HPV risk, and diagnosis, showed a large effect size. In other words, the sample size of the four diagnosis groups had a low probability of introducing statistical bias on the results.

**Figure 3:**
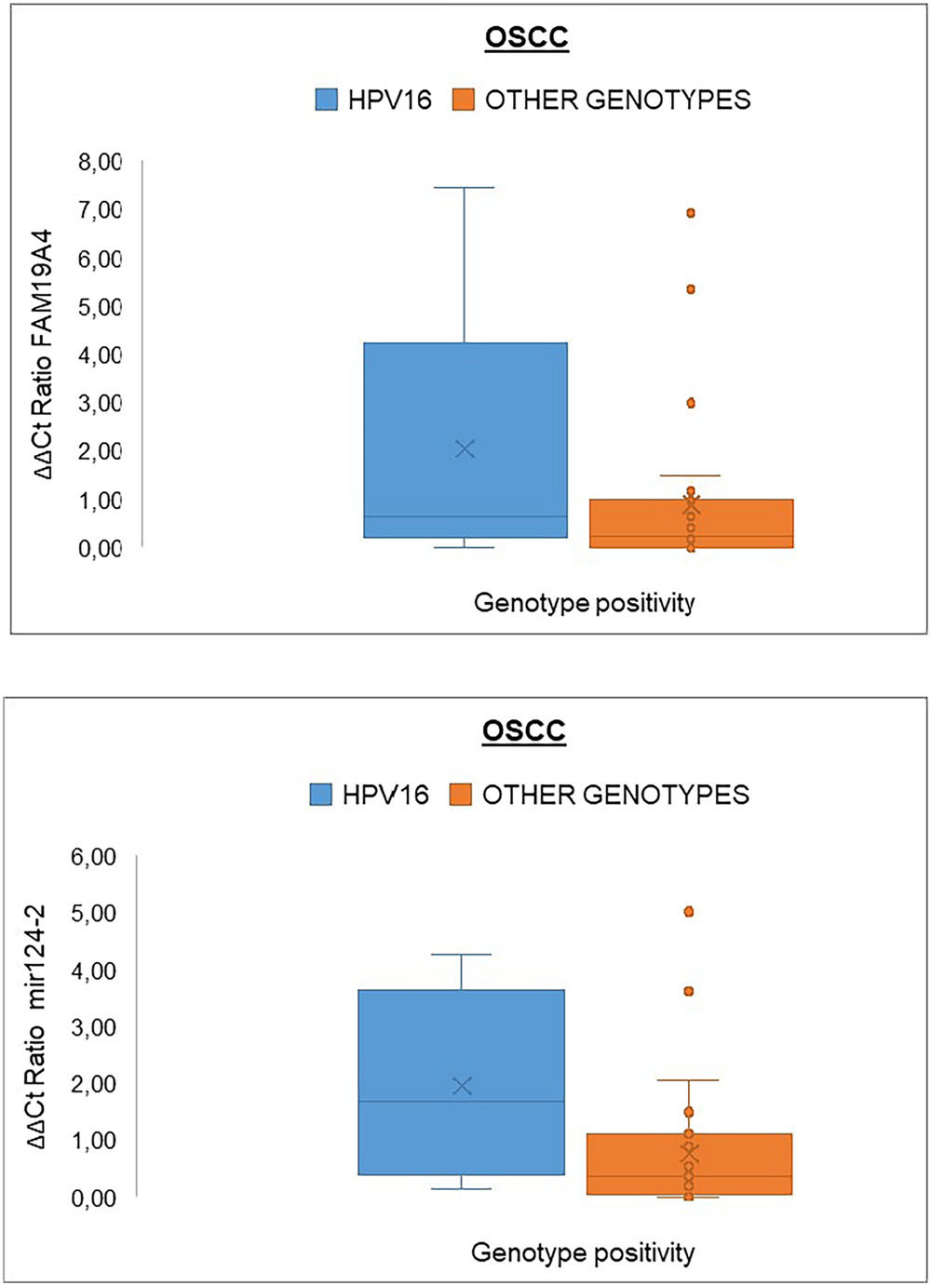
Box plots showing the ΔΔ*Ct* ratio values of *FAM19A4* and *miR124‐2* in OSCC samples positive for HPV16 compared to samples positive for all the other genotypes. OSCC, oral squamous cell carcinomas.

Finally, the HPV16‐positive OSCCs were specifically selected to evaluate whether their methylation levels were higher than the other HPV‐positive samples. Figure 3 shows that HPV16 was associated with the hypermethylation of both targets.

## 4 Discussion

Virus‐induced carcinogenesis represents a significant burden in the global public health scenario, with over 10% of tumors caused by viral infections [45]. Harald Zur Hausen first defined the role of HPV as an oncovirus, and since then its involvement in the initiation and development of tumors has been extended from cervical carcinoma to several other malignancies [46].

The last challenge in this regard is represented by HPV‐related HNSCCs, particularly those in the oropharynx and oral cavity, which appear to have met a significant increase since the nineties, especially in high‐income countries like the United States and the Scandinavian peninsula [47]. However, the epidemiology of oral HPV infection and the natural history of the virus in the oral cavity are still poorly understood, leading to delayed or under‐diagnoses [48]. Adding to the complexity of the diagnosis are the potentially malignant lesions, which are not per se clear precursors of oral neoplastic lesions, and whose association with HPV infection is far from being defined [23].

Untangling the complex skein of cancer development has led to the identification of molecular biomarkers, which help define the diagnosis, prognosis, and treatment of malignancies. In this regard, the research on cervical carcinoma has produced interesting and promising results, leading to the placement in the market of diagnostic kits designed to identify the hypermethylation of two targets, *miR124‐2* and *FAM19A4*.

This approach can efficiently detect 98.3% of cervical cancers and improve triage of hrHPV‐positive patients: patients with hypermethylation are at risk of developing cervical neoplasm, while patients without hypermethylation have a low cancer risk that also extends to 14 years [34].

To our knowledge, this is the first study to analyze *miR‐124‐2* and *FAM19A* methylation levels oral rinses belonging to OSCC, OPMD, and BL patients. Oral rinses are a well‐established method for diagnosing oral HPV infection, not to mention much less invasive and perhaps just as informative as tissue samples. This approach has already been successfully used by Pauciullo et al. [49] to assess the methylation levels of the two targets in HIV‐positive men who have sex with men.

Hypermethylation results were observed using two different approaches: an analysis performed under the lens of the hypermethylation positivity cut‐off developed for cervical samples, in parallel with a ΔΔ*Ct* ratio‐dependent analysis. Both ways painted a picture highly suggesting the involvement of hypermethylation in the progression toward oral cancer.

In the first case, presence of hypermethylation in the total sample, that is, one of the two target ΔΔ*Ct*s below the cervix‐defined cut‐offs, was significantly more frequent in cancerous lesions (*p* < 0.0001), whereas absence hypermethylation was significantly more frequent in patients without lesions and with BL (*p* = 0.0023 and *p* = 0.0162, respectively). This approach was used by Pauciullo et al. [49] who analyzed HIV‐positive MSM with oral HPV infection but no history of oral cancer. The study highlighted that 90.91% of methylated samples were associated with hrHPV, suggesting a potential role in future screening programmes.

As for ΔΔ*Ct* Ratio analysis, it showed, again, how the two targets, taken together or separately, are highly methylated in oral cancer samples and, in addition, the level of their hypermethylation increases progressively from patients with no diagnosed lesions to those with BL, OPMDs, and OSCCs. In other words, the analysis of hypermethylation percentages alone allows a clear differentiation between the four groups and their diagnosis.

These findings suggest a possible prognostic role of *FAM19A4* and *miR124‐2* methylation assessment: patients with BL and, especially, OPMDs showing presence of hypermethylation for one or both targets should receive close follow‐up.

As cervical cancer has a 99.7% correlation rate with HPV infection, the assessment of the two target methylation levels in an HPV‐negative scenario has not been evaluated in the literature. The same cannot be said for oral cancer, whose association with persistent HPV infection amounts to much lower rates: from the 10.6% figure reported by the National Cancer Database (NCDB) [50] to the broad range of 0%–37% described in the recent meta‐analysis by Katirachi et al. [15].

The cohort analyzed, where the sample selection was specifically designed to have approximately equal numbers of HPV‐positive and HPV‐negative samples in each group, allowed a separate hypermethylation analysis in HPV‐positive and HPV‐negative samples.

In particular, the significance of the correlation between hypermethylation and OSCC diagnosis was maintained in both cases: HPV‐positive samples were significantly correlated with hypermethylation (*p* = 0.0006), like HPV‐negative ones, which showed a correlation *p*‐value of 0.0007 with hypermethylation.

These data were complemented by the finding that in the NL group, when the virus could not be found, the same was true for hypermethylation (*p* = 0.0105).

These pieces of evidence contributed to the assumptions that the methylation of the two targets is not strictly correlated with HPV infection but may be one of the epigenetic alterations involved in the progression to oral cancer.

Literature findings describe how aberrant methylation of *miR124‐2* and *FAM19A4* promoters is a phenomenon that could characterize the neoplastic evolution per se, without the intervention of a persistent viral infection. Indeed, *FAM19A4* was one in a multitude of genes analyzed through WGBS whose corresponding datasets can be found on the *Gene Expression Omnibus* (GEO) platform under the following accession numbers GSE38532 [51, 52], GSE41114 [51], GSE46802 [39, 53]. These studies showed a *FAM19A4* promoter's statistically significant hypermethylation in oral cancer tissue biopsies compared to adjacent noncancerous tissue [39, 51, 52, 53].

There is talking specifically about Sheu et al. [51], and Lee et al. [52], which analyzed tumor and nontumor pair‐wise samples of a cohort of OSCC patients exposed to risk factors such as alcohol, areca nut and smoke, with the WGBS (Whole Genome Bisulphite Sequencing) platform Illumina HumanMethylation27 BeadChip (HumanMethylation27_270596_v.1.2). Among the tested targets, the CpG island located upstream from *FAM19A4* gene showed a significant difference in methylation between cancerous and healthy tissue with adjusted *p*‐values of 1.70e^−27^, 1.04e^−26^.

Using the same WGBS platform, Towle et al. [39, 53] tested 30 biopsies corresponding to dysplastic, in situ carcinoma (CIS), OSCC, and adjacent normal tissues, obtained from 10 patients undergoing surgical removal of OSCC or CIS. Different methylation patterns were found between the normal and the CIS/OSCC tissues, with *FAM19A4* counted among the genes showing a significant alteration in the methylation level (*p* = 1.01e^−04^, 4.34e^−04^).

*Mir124‐2*, on the other hand, does already have a clearly defined role as a tumor suppressor, which manifests through the inhibition of proliferation, aggressiveness, apoptosis, and invasion of tumor cells [54]. In general, it has been shown that microRNA genes are several times more often methylated than protein‐coding genes, and regarding *mir124‐2*, its methylation‐dependent silencing has already been analyzed and defined in several neoplasms that go beyond HPV‐related cervical cancer, that is, ovarian, vulvar, breast, lung, and colorectal cancers [40, 55, 56, 57, 58].

For example, *mir124‐2* has been found by Pangeni et al. [59] as one of the epigenetically dysregulated genes in breast to brain metastases compared to the primary breast tumor.

A different result was described by Oltra et al. [60], in whose study *miR124‐2* hypomethylation and correspondingly higher expression were associated with poor overall survival in young women (< 35 years) with breast cancer. This result, in contrast to the one described in older women, where hypermethylation is associated with a worse prognosis, could suggest a possible role as a specific prognostic factor.

Studies on HPV have often highlighted the different aberrant dynamics induced by the distinct HPV genotypes. These differences lie in the peculiar molecular characteristics identified between cutaneous and mucosal types, lr and hrHPVs, and between individual high‐risk types. The main differences concern the two oncoproteins, E6 and E7, which influence carcinogenic potential and can interfere with methyltransferase activity [61].

Since the two oncoproteins are responsible for the impaired methylation scenario, it can be assumed that the aberration in methylation is induced differently depending on the HPV risk classification.

The ΔΔ*Ct* Ratio approach was used to assess whether the hypermethylation was indicative of the association between hr or lrHPV genotypes and the diagnosis: statistically significant data emerged in lrHPV positive samples only for *FAM19A4*, whose ΔΔ*Ct* ratio scores were, surprisingly, higher in OSCC group than BL group (mean: 0.743 vs. 0.083, *p* < 0.05).

Speaking specifically about positive OSCCs, the separate analysis of *FAM19A4* and *miR124‐2* methylation percentages in HPV16‐positive OSCC samples compared to those positive for other genotypes revealed a visually striking difference. Indeed, hypermethylation was significantly higher when HPV16 was detected.

HPV16 is undoubtedly the genotype whose involvement in tumorigenesis has been better characterized, being the most frequently identified genotype in cervical, anal, penile, oropharyngeal and oral cancers [62]. Even if some authors [63, 64, 65] still question the causal relationship between infection and oral neoplasia, the evidence of HPV16 association with higher methylation levels indicates a possible peculiar methylation and molecular landscape, and thus further suggests the actual involvement of HPV, at least HPV16, in oral carcinogenesis.

The detection of genotypes belonging to the betapapillomavirus genus (e.g., HPV120) raises questions about the role of these cutaneous subtypes in oral disease. Classically associated with the occurrence of warts on the hands and feet, betapapillomaviruses are poorly studied, but our work was not the first to highlight their presence in the oral cavity. Sabol et al. [66], looking for beta‐HPV in oral rinses and tissue biopsies from patients with oral cancer, oral lesions, and healthy patients, found a striking overall prevalence of 54.8%, as well as a strong association between HPV5, HPV122, and oral cancer. However, the poor concordance between the presence of the virus in mouthwash and in tumor tissue makes the authors skeptical about the causal role of beta genotypes in carcinogenesis, suggesting instead a role as an environmental or infectious co‐factor. Similar findings were reported by Agalliu et al. [67] who found an increased risk of cancer associated with beta HPV infection.

Doubts about the etiological role of betapapillomaviruses in the development of oral neoplasia are reported in the case‐control study by Al‐Sonedair et al. [68], where a higher prevalence was found in the control group, and lower odds of β‐HPV infection were found among cases.

In conclusion, this study has shown for the first time how assessment of hypermethylation of two targets, the *FAM19A4* and *mir124‐2* promoters, can aid in the diagnosis of OSCC and, more importantly, identify those patients affected by OPMDs at risk of cancer progression. To prove the exact prognostic value of hypermethylation assessment in the OPMD context will require a close follow‐up of patients, as well as the design of future longitudinal studies.

The relatively low number of patients, especially HPV positives, did not enable bringing out the statistically significant differences in the effect of HPV infection on targets’ methylation level.

Therefore, increasing the sample size to confirm these preliminary results and outline new significant information is imperative.

The future of this topic would certainly include studies of methylation‐related changes in transcriptomic and proteomic pathways. This would allow an understanding of the biomolecular processes affected by aberrant methylation of *FAM19A4* and *mir124‐2* and thus directly involved in the progression to the cancer phenotype.

## Author Contributions

**Michela Buttà:** conceptualization, methodology, investigation, data curation, writing – original draft, writing – review and editing, visualization, supervision. **Nicola Serra:** conceptualization, methodology, software, formal analysis, data curation, writing – original draft, visualization. **Arianna Sucato:** methodology, investigation, visualization. **Daniela Cabibi:** methodology. **Giuseppina Campisi:** methodology. **Vera Panzarella:** investigation. **Giulia Alfedi:** methodology, software. **Daniela Pistoia:** investigation. **Giuseppina Capra:** conceptualization, methodology, data curation, investigation, writing – review and editing, visualizzation, supervision, resources, project administration.

## Ethics Statement

Approval numbers #03/2013 and #04/2024.

## Consent

Informed written consent was obtained from each subject. The study was performed at the University Hospital “P. Giaccone.”

## Conflicts of Interest

The author, Giulia Alfedi, declares to be an employee of the company Fujirebio Italia. This employment relationship has not influenced in any way the contents of this manuscript, which was written in complete scientific autonomy. The remaining authors declare no conflicts of interest.

## Supporting information

Supporting material 1.

Supporting Material 2.

## Data Availability

Data sharing is not applicable to this article as no new data were created or analyzed in this study.
