# Supplementary material for: The Role of Methylation as an Epigenetic Marker in HPV‐Related Oral Lesions
Source: J Med Virol. 2025 Jun 24;97(7):e70459. doi: 10.1002/jmv.70459 (PMC12188162; doi:10.1002/jmv.70459)
Supplement: Supplementary file 1 — Supporting material 1. [file JMV-97-e70459-s001.docx]

**Supplemental material 1**. Clinical information: age, sex, diagnoses, and

infection type, in the total sample group.

| **Parameters** | **Sample data** |
| --- | --- |
| *Patients* | 111 |
| *Age* | 55.7 ± 16.2 |
| *Sex* |  |
| Male | 60.4% (67) |
| Female | 39.6% (44) |
| *Diagnoses* |  |
| NL | 18.0% (20) |
| BL | 33.3% (37) |
| OPMD | 15.3% (17) |
| OSCC | 33.3% (37) |
| *HPV infected* | 48.6% (54) |
| *Number genotypes* |  |
| One | 81.5% (44) |
| Two | 7.4% (4) |
| Three | 3.7% (2) |
| More than three | 7.4% (4) |
| *Genotype risk* |  |
| hr/hr-lr | 64.8% (35) |
| lr | 35.2% (19) |
| *Hypermethylation* |  |
| Absence | 65.8% (73) |
| Presence | 34.2% (38) |

NL = No Lesions; BL = Benign lesions; OPMD = potentially malignant

lesions; OSCC = Cancer lesions; lr= low risk; hr= high risk.
