# Supplementary material for: The Role of Methylation as an Epigenetic Marker in HPV‐Related Oral Lesions
Source: J Med Virol. 2025 Jun 24;97(7):e70459. doi: 10.1002/jmv.70459 (PMC12188162; doi:10.1002/jmv.70459)
Supplement: Supplementary file 2 — Supporting Material 2. [file JMV-97-e70459-s002.doc]

**Supplementary Material 2.** Stratification of the groups NL, BL, OPMD and OSCC according to presence and absence of hypermethylation

| **Parameters** | **NL-Age**  **n=20** | **BL-Age**  **n=37** | **OPMD-Age**  **n=17** | **OSCC-Age**  **n=37** |
| --- | --- | --- | --- | --- |
| Hypermethylation |  |  |  |  |
| yes | 55 ± 0.0  5% (n=1) | 57.7 ± 7.1  18.9% (n=7) | 73.2 ± 8.6  35.3% (n=6) | 65.7 ± 12.8  64.9% (n=24) |
| no | 38.6± 9.7,  95% (n=19) | 49.5± 16.7  81.1% (n=30) | 62.7± 10.1  64.7% (n=11) | 62.2± 11.5  35.1% (n=13) |
| Statistical analysis  yes vs no | Cannot  perform test | p=0.22 (T) | p=0.0487* (T) | p=0.41 (T) |
| Power analysis |  | *d* = 0.53  medium effect | *d* = 1.09  large effect | *d* = 0.29  Low effect |
| *=significant test; no lesions (NL), benign lesions (BL), oral potentially malignant disorders (OPMD) and oral squamous cell carcinoma (OSCC). | | | | |
